# Ozone water enema activates SIRT1-Nrf2/HO-1 pathway to ameliorate gut dysbiosis in mice receiving COVID-19 patient-derived faecal microbiota

**DOI:** 10.1099/jmm.0.002038

**Published:** 2025-09-17

**Authors:** Zehua Su, Jiaqi Lin, Xuejiao Zeng, Xin Li, Qianhao Hou, Qing Wang, Chunzheng Liu, Jiawen Qin, Yuling Li, Jinyuan Zhang, Xiangrui Wang, Shuwen Qian, Lijun Liao

**Affiliations:** 1 Department of Pain Management, School of Medicine, Shanghai East Hospital Affiliated To Tongji University, Shanghai 200120, PR China; 2 Department of Anesthesiology and Pain Management, School of Medicine, Yangpu Hospital Affiliated To Tongji University, Shanghai 200090, PR China

**Keywords:** COVID-19, gut microbiota, Nrf2 (nuclear factor erythroid 2-related factor 2)/HO-1 (heme oxygenase-1), ozonated water enema, SIRT1(silent information regulator of transcription 1)

## Abstract

**Introduction.** This study centres on how coronavirus disease 2019 (COVID-19) disrupts the intestinal microbiota and amplifies systemic inflammation and evaluates ozone water enemas as a strategy to restore gut microbial balance and activate the SIRT1 (silent information regulator of transcription 1)-Nrf2 (nuclear factor erythroid 2-related factor 2)/HO-1 (heme oxygenase-1) pathway for alleviating post-viral sequelae. Our findings demonstrate that ozone water intervention markedly improves the intestinal microenvironment in mice receiving COVID-19 patient-derived microbiota and attenuates systemic inflammation, offering a viable adjunctive approach for COVID-19 management.

**Hypothesis.** Despite significant progress in reducing the incidence of COVID-19, its long-term consequences, including hepatic dysfunction, pulmonary injury and gut microbiota dysbiosis, remain challenging. While ozonated water enema therapy has shown efficacy in alleviating inflammation and neutralizing oxidative stress, the precise mechanisms by which ozonated water attenuates COVID-19 progression are not fully understood. We hypothesized that ozonated water enemas could enrich gut microbiota composition in COVID-19 patients, thereby optimizing the gut environment following faecal transplantation in a murine model.

**Aim.** The overarching aim of this investigation was to ascertain whether ozonated water enemas could exert a salutogenic effect on the gut microbiota in a mouse model, as well as on the holistic gut and systemic health of critically ill COVID-19 patients subsequent to faecal transplantation.

**Methodology.** The entire experiment was conducted over a 14-day period. WT mice were randomly allocated into three groups: Sham, FMT (faecal microbiota transplantation) and FMT+O_3_ (FMT with ozonewater enema treatment). Mid-stage faecal specimens were collected from 21 severe COVID-19 patients and randomly divided into seven subgroups (three specimens per subgroup). These specimens were transplanted into the WT mice of the FMT and FMT+O_3_ groups via faecal gavage on days 1 through 7. The healthy control group (Sham) received oral administration of ddH₂O instead. Starting on day 8 post-transplantation, the FMT+O_3_ group underwent ozone water enema treatment for seven consecutive days. During this treatment period, assessments were performed to evaluate intestinal barrier function, inflammatory changes and alterations in gut microbiota. Additionally, improvements in intestinal, hepatic, pulmonary and systemic lesions were examined.

**Results.** Our findings indicate that ozonated water enemas modulate the SIRT1-Nrf2/HO-1 pathway, significantly enhancing the intestinal environment in mice that received FMT from COVID-19 patients. This intervention increased microbiota populations, strengthened intestinal barrier integrity and reduced intestinal and systemic inflammatory responses.

**Conclusion.** The results highlight the potential of ozonated water enemas as a therapeutic option for COVID-19 patients, particularly in optimizing intestinal microbiota and mitigating inflammatory responses through SIRT1-Nrf2/HO-1 pathway modulation. This approach offers a novel strategy for addressing residual effects of COVID-19.

## Data Summary

Datasets for this current study can be found under BioProject number PRJNA1304050. Individual sample accession numbers can be found in Table S2, available in the online Supplementary Material.

## Introduction

Coronavirus disease, instigated by the severe acute respiratory syndrome coronavirus 2 (SARS-CoV-2), manifests as a highly contagious respiratory ailment [1]. The pathogenesis of this disease entails the virus binding to the angiotensin-converting enzyme 2 (ACE2) receptor within the respiratory tract, thereby enabling viral ingress into host cells. This pivotal step precipitates viral replication and the subsequent release of pro-inflammatory cytokines [2]. In severe cases of coronavirus disease 2019 (COVID-19), patients may succumb to acute respiratory distress syndrome (ARDS), sustain pulmonary damage and experience multiorgan failure [3]. The ramifications of the disease are far-reaching, encompassing widespread morbidity and mortality, economic upheaval, overwhelming strain on healthcare systems and critical shortages of medical supplies and personnel. Public health interventions such as lockdowns, social distancing mandates and stringent travel restrictions have profoundly impacted daily living and the global economic landscape. Amidst these challenges, the clinical imperative persists in the pursuit of antiviral therapies and the comprehensive management of systemic symptoms and sequelae that arise in the wake of novel coronavirus outbreaks [4,5].

Wang *et al*. showed that the gastrointestinal tract is the largest immune organ in the human body [6]. The human gut microbiota, comprising over 100 trillion micro-organisms including bacteria, archaea, fungi and viruses, plays a crucial role in digestion, metabolism, immune function and protection against pathogens [7]. Owing to its pivotal role in digestion and metabolism, the intestinal microbiota has earned the moniker of the ‘second brain’ [8]. Latest investigations have revealed that COVID-19 can perturb the composition of the intestinal microbiota, compromise gut barrier integrity, facilitate the translocation of bacterial endotoxins into the circulatory system and amplify systemic inflammatory reactions [9,10]. An imbalance in the gut microbiota, or dysbiosis, may impede the mobilization of immune cells across the body, thereby increasing vulnerability to a spectrum of diseases. Consequently, examining the gut microbiome is essential for understanding the development of novel coronavirus infections and evaluating the severity and prognosis in patients. In summary, gut bacteria and their metabolites significantly influence our overall health and immune system, highlighting the need for more research on their impact on infectious diseases like COVID-19.

Research indicates that COVID-19 patients exhibit reduced diversity in intestinal microbiota [11,12]. This reduction is attributed to viral invasion, which instigates the activation of pattern recognition receptors, thereby prompting innate immune cells to secrete pro-inflammatory mediators [13]. As a result, this cascade of events undermines the integrity of the intestinal barrier and disrupts the equilibrium of the bacterial microbiota, leading to a proliferation of pathogenic bacteria and a concomitant decline in beneficial microbial species [13]. Beyond gastrointestinal symptoms such as nausea and vomiting [14], severe manifestations of COVID-19 can induce substantial perturbations in the intestinal microecology. These disturbances in the gut microbiota can potentially precipitate systemic complications. Although these findings are preliminary, they collectively underscore gut dysbiosis as a critical nexus linking localized viral pathology to systemic sequelae. Critically, restoring microbial balance through targeted interventions could mitigate both intestinal and extra-intestinal manifestations of COVID-19: a rationale that directly informs our investigation of ozone therapy.

There is increasing interest in the potential of ozone therapeutic treatment to alleviate systemic symptoms across various conditions. Administration of ozonated water through rectal enema is considered an invasive but promising treatment. It is straightforward to administer and is believed to be safe due to its supposed ability to modulate oxidative stress, decrease inflammation and enhance tissue oxygenation [15]. Studies have demonstrated that ozone therapeutic treatment can exert systemic effects by influencing the abundance of the gut microbiota, enhancing antioxidant capacity and modulating the immune response [16,18]. For instance, Cenci *et al*. showed that ozone can trigger cell excitation responses through reactive oxygen species (ROS) as signalling molecules, leading to the activation of cellular survival mechanisms such as the Nrf2 (nuclear factor erythroid 2-related factor 2)/Keap1/ARE system and the AMPK/FOXO/mTOR/Sir1 pathway [19]. This, in turn, can influence the gut microbiota and immune response, potentially affecting the progression of diseases. Clinical cases have also shown the effectiveness of this approach in ameliorating systemic symptoms in COVID-19 patients [20,22]. Furthermore, the safety of ozonated water enemas has been established in clinical settings, providing a foundation for exploring their use as an adjunctive therapy for managing systemic manifestations in various disease states [18,23]. This suggests that ozonated water enemas could be a valuable addition to the therapeutic arsenal for managing not only COVID-19 but also other conditions where systemic symptoms are prevalent. The SIRT1 (silent information regulator of transcription 1)-Nrf2/HO-1 (heme oxygenase-1) pathway is associated with various diseases [24,25]. SIRT1, a key regulator of cellular homeostasis, interacts with the Nrf2/HO-1 pathway to modulate antioxidant and anti-inflammatory responses [26,27]. In COVID-19 patients, oxidative stress and inflammation are common, and activating this pathway can help reduce these effects [28]. The gut microbiota, influencing systemic inflammation and immune responses, is also affected by this pathway [29,30]. Ozone-water enemas can activate the SIRT1-Nrf2/HO-1 pathway through mild oxidative stress, enhancing the body’s antioxidant capacity and reducing inflammation [31,32]. By targeting this axis, ozone enemas may not only rehabilitate the gut microenvironment but also disrupt the gut-systemic inflammatory loop perpetuating COVID-19 complications, providing a mechanistic basis for our hypothesis.

However, the mechanism by which ozonated water enemas improve the intestinal microbiota and reduce systemic complications and sequelae in COVID-19 patients is not yet well understood. Therefore, we investigated the potential benefits of administering ozonated water enemas to mice that received faecal microbiota transplants from COVID-19 patients, aiming to enhance their intestinal microbiota and attenuate systemic inflammatory responses. Our study highlights the impact of COVID-19 pathogenesis on the entire body, particularly the intestinal microenvironment, and offers theoretical foundations and alternative treatment avenues for COVID-19.

## Methods

### Subject recruitment and sample collection

This study followed the principles outlined in the Declaration of Helsinki, and written informed consent was obtained from all participants. Approval for the study was granted by the Medical Ethics Committee of Shanghai East Hospital (no. 2022202). Between December 2022 and January 2023, we enrolled 21 patients with COVID-19 from the Respiratory Critical Care Emergency Department of Shanghai East Hospital. Inclusion criteria for stool sample collection included a positive test for coronavirus antigen, the presence of at least four respiratory complications (e.g. fever, persistent cough, shortness of breath, respiratory tract infections and pneumonia), SaO2≤90% or PaO2≤60 mmHg, no haemolysis, body mass index between 18.5 and 24, aspartate transaminase (AST) and alanine aminotransferase (ALT) values≥20 mmHg and no haemorrhagic fever. Faecal samples were collected in the hospital, mixed with cold PBS (4 °C) at a 1 : 3 ratio. Samples were refrigerated at 4 °C before faecal microbiota transplantation (FMT), and the trial’s exclusion criteria included underlying respiratory diseases (e.g. chronic obstructive pulmonary disease), chronic gastroenteritis, history of gastrointestinal surgery, psychiatric disorders, Parkinson’s disease, diabetes and pregnancy.

### Animals and treatments

Specific pathogen-free male C57BL/6J mice (16 weeks old, 25–30 g; Shanghai Centre for Model Organisms) were housed under a 12 h light–dark cycle with *ad libitum* access to food and water. The mice (*n*=10/group) were randomly assigned to three experimental cohorts:

Sham: oral gavage with PBS on days 1–7, followed by double-distilled water (ddH_2_O) enema on days 8–14.

FMT: FMT via oral gavage on days 1–7 [33,35], then ddH2O enema on days 8–14.

FMT+O_3_: FMT identical to group 2 during days 1–7, followed by ozonated water (30 µg ml^−1^ [36,38]) enema on days 8–14.

FMT preparation: fresh faecal samples from 21 critically ill COVID-19 patients were pooled into seven donor groups assigned to days 1–7 (3 patients/group). Then, on days 1–7 of the experiment, faeces from the group of the day (3 patients) were collected, mixed well and homogenized in PBS at a ratio of 1 : 3 (w/v), vortexed for 5 min and then left to stand (30 min, 4 °C). The supernatant was filtered through a 70 µm cell strainer and administered (100 µl/mouse) within 2 h post-preparation.

Ozonated water administration: on days 8–14, FMT+O_3_ mice were anesthetized with 3% sevoflurane (10 min exposure), followed by insertion of a polyethylene catheter (4 cm depth from anal verge). Ozonated water (200 µl/mouse) was infused slowly using a 1 ml syringe. To ensure uniform colonic distribution, mice were maintained in a vertical inverted position for 10 min post-administration before returning to standard housing.

Terminal sampling: all mice underwent terminal blood/tissue collection under sevoflurane anaesthesia at day 14, 8 h after the final intervention. After sacrifice, tissues were rapidly excised using sterile surgical instruments. The collected tissues were immediately placed in pre-chilled collection tubes containing appropriate buffers or fixatives to prevent degradation and maintain sample integrity. For RNA isolation, tissues were snap-frozen in liquid nitrogen and stored at −80 °C until processing with TRIzol. For histological analysis, tissues were fixed in 10% neutral buffered formalin immediately after collection, processed through routine paraffin embedding, sectioned and then subjected to staining procedures as described.

### Experimental design and bias control

Randomization protocol: mice were allocated to treatment groups (Sham, FMT and FMT+O_3_) using a stratified randomization approach to ensure baseline homogeneity:

Step 1: All mice (*n*=30) were initially grouped by body weight (25–30 g range) into three strata (25–26.5, 26.6–28.5 and 28.6–30 g).

Step 2: Within each stratum, mice were randomly assigned to the three treatment groups using a computer-generated random number sequence (GraphPad Prism v9.0).

Step 3: Final group assignments were validated to ensure no significant differences in baseline weight (one-way ANOVA, *P*=0.76) or age (Kruskal–Wallis test, *P*=0.82).

Blinding during outcome assessment: to minimize observer bias, a double-blind design was employed:

Intervention phase: personnel administering FMT or ozonated water enemas were unaware of group assignments. Treatments were coded as ‘A’, ‘B’ and ‘C’ (decoded post-analysis).

Endpoint measurements: objective parameters (e.g. serum cytokines, faecal 16S sequencing and oxidative stress markers) and semi-quantitative assessments (e.g. histopathology scoring).

### Real-time PCR

Total RNAs were isolated from tissues using TRIzol reagent (Thermo Fisher Scientific, USA), and cDNA was synthesized using the ChamQ Universal SYBR qPCR Master Mix (Vazyme, China). The qPCR procedure involved an initial 30 min incubation at 95 °C, followed by 40 cycles of 10 s at 95 °C and 30 s at 60 °C. SYBR Green System (Yeasen, Shanghai) was utilized for qPCR according to the provided instructions. The relative gene expression was calculated using the 2−ΔΔCt method, with GAPDH serving as an internal control and reference gene. The primers used in the study are listed in Table 1.

**Table 1.** Primer sequences used for quantitative real-time PCR in this study

	Forward primers	Reverse primers
GAPDH	TCAAGCTCATTTCCTGGTATGAC	CTTGCTCAGTGTCCTTGCTG
CD45	GAACATGCTGCCAATGGTTCT	TGTCCCACATGACTCCTTTCC
ZO-1	TGAACGTCCCTGACCTTTCG	CTGTGGAGACTGCGTGGAAT
IL-1β	TGCCACCTTTTGACAGTGATG	TTCTTGTGACCCTGAGCGAC
Muc2	GCTGACGAGTGGTTGGTGAATG	GATGAGGTGGCAGACAGGAGAC
F4/80	TTTCCTCGCCTGCTTCTTC	CCCCGTCTCTGTATTCAACC
HO-1	GAATCGAGCAGAACCAGCCT	GCCTTCTCTGGACACCTGAC
NLRP3	CCACAAGATCGTGAGAAAACCC	CGGTCCTATGTGCTCGTCA
Nrf2	AAGAATAAAGTCGCCGCCCA	AGATACAAGGTGCTGAGCCG

### Immunohistochemical staining

Sampled tissues were fixed in 10% neutral formalin for 24 h and then dehydrated progressively in graded alcohols, with 1 h of immersion at each concentration, followed by hyalinization in xylene before paraffin embedding using an embedding machine. Three consecutive sections of each sample were stained, and the whole sections were analysed to ensure a thorough assessment and to obtain a representative and complete picture of the staining pattern of the whole tissue. Tissue paraffin sections with a thickness of 5 µm were first placed in an oven at 65 °C for 45 min, then deparaffinized and rehydrated. Antigen retrieval was achieved by heating sections in citrate buffer (pH 6.0) using an autoclave. Tissue sections were then incubated in 3% H_2_O_2_ for 15 min, followed by a PBS rinse (5 min, 3 times) and then incubated with 5% BSA at 25 °C for 30 min. The sections were then incubated with monoclonal antibodies against CD45 (1 : 500, Cell Signaling), CD11b (1 : 700, Cell Signaling) [39], F4/80 (1 : 1,000, Cell Signaling) [39] and NLRP3 (1 : 700, Immunoway) at 4 °C overnight. After rinsing with PBS (5 min, 3 times), the sections were incubated with polymerized horseradish peroxidase-labelled goat anti-rabbit IgG (1 : 1,000, Beyotime) for 40 min at 37 °C. Detection was performed by DAB staining (Vector Labs), and sections were counterstained with haematoxylin. Finally, the sections were dehydrated, cleared and fixed with neutral gel.

Positive and negative controls were used in each experiment to confirm staining specificity. For negative controls, sections were incubated with isotype-specific immunoglobulins at the same protein concentration as the primary antibody, ensuring that any observed staining was due to specific binding rather than nonspecific interactions [40].

### Immunofluorescence staining

After the collected tissue samples were immediately embedded in Tissue-Tek, using a cryotome, the tissues were cut into 6-µm-thick sections and stored at −80 °C. Slides were air-dried for 30 min at room temperature followed by 4% polyfluoroalkoxy fixation. Next, tissue samples were blocked for 1 h at room temperature in a humidity chamber containing 5% goat serum. After blocking, samples were incubated with the primary antibody zonula occludens-1 (ZO-1) (1 : 200, Abcam) and left in a humidity chamber at 4 °C overnight. Samples were washed thoroughly in PBS and then incubated with the secondary antibodies for 1 h in a humidity chamber. After incubation, sections were thoroughly washed again in PBS. Finally, the sections were mounted in an aqueous DAPI medium (Life-iLab) to counterstain nuclei. Mucus staining was performed according to an established protocol [41]. Briefly, colon tissue sections containing faeces were fixed using the Carnoy fixation method (60% absolute methanol, 30% chloroform and 10% glacial acetic acid). After paraffin embedding, mucus and intestinal bacteria were stained with an anti-Mucin-2 primary antibody (1 : 1,000, Santa Cruz).

### Western blot

The total proteins in the tissues were first extracted using RIPA lysis buffer (Epizyme Biotech, China) supplemented with a 1% protease inhibitor cocktail (Epizyme Biotech, China). The protein concentration was determined by centrifugation at 4 °C and 12,000 r.p.m. (16,000 ***g***) for 15 min, and the bicinchoninic acid assay kit (Solarbio, China) was used for detection. Subsequently, the samples were boiled at 100 °C for 5 min, and then, the proteins were separated on a 12.5% gel prepared using the SDS-PAGE Gel Fast Preparation Kit (Epizyme Biotech, China) for electrophoretic separation and immunodetection. Electrophoresis was followed by transfer to a PVDF membrane (Millipore, Burlington, MA, USA) at a constant current of 0.25 A for 90 min. The membranes were blocked with 5% nonfat milk-TBST solution for 1 h and then incubated overnight at 4 °C with the following primary antibodies: rabbit anti-GAPDH (1 : 5,000, Proteintech), rabbit anti-P-P-65 NF-κB (1 : 1,000, Wanleibio), rabbit anti-P-65 NF-κB (1 : 1,000, Wanleibio), rabbit anti-caspase-1 (1 : 1,000, Proteintech), rabbit anti-interleukin-1β (IL-1β; 1 : 1,000, Abcam), rabbit anti-Nrf2 (1 : 200, Santa Cruz) and rabbit anti-SIRT1 (1 : 1,000, Proteintech). Protein bands were detected with HRP-conjugated rabbit secondary antibody (1 : 5,000, Beyotime). After incubation with the secondary antibody, specific bands were visualized using chemiluminescence imaging on a ChemiDoc XRS+ System (Bio-Rad, USA) with an ECL kit (Juhemei, China). Protein bands were analysed using ImageJ software.

### Sample collection and testing

On day 14 of the experiment, faecal samples were collected from 21 mice. Total genomic DNA was extracted from these samples using the CTAB method. The concentration and purity of the DNA were assessed on 1% agarose gels, which are ideal for separating DNA fragments ranging from 500 bp to 10 kb. Based on these assessments, the DNA was diluted to a concentration of 1 ng µl^−1^ with sterile water. For the amplification of the 16S rRNA genes (16S V3–V4 regions), specific primers (341F: 5′-CCTAYGGGRBGCASCAG-3′ and 806R: 5′-GGACTACNNGGGTATCTAAT-3′) with barcodes were used. Each PCR reaction was performed using 15 µl of Phusion^®^ High-Fidelity PCR Master Mix (New England Biolabs), 2 µM of both forward and reverse primers and ~10 ng of template DNA. The thermal cycling conditions consisted of an initial denaturation at 98 °C for 1 min, followed by 30 cycles of denaturation at 98 °C for 10 s, annealing at 50 °C for 30 s and elongation at 72 °C for 30 s. A final extension step was performed at 72 °C for 5 min. To analyse the PCR products, equal volumes of 1× TAE buffer were mixed with the PCR products, and electrophoresis was performed on 2% agarose gels, which provide better resolution for smaller DNA fragments due to their smaller pore size. The PCR products were then mixed in equidensity ratios. The mixture of these fragmented PCR products was purified using the Universal DNA Purification Kit (Tiangen, China).

### Libraries generated and Illumina sequencing

Sequencing libraries were generated using the NEBNext^®^ Ultra DNA Library Prep Kit (Illumina, USA) following the manufacturer’s protocol, with index codes added for sample identification. The quality of the libraries was assessed using the Agilent 5400 system (Agilent Technologies Co. Ltd., USA) to ensure proper fragment size distribution and concentration. The validated libraries were then sequenced on the Illumina platform, generating 250 bp paired-end reads for subsequent bioinformatics analysis.

### Bioinformatics analysis

The analysis was conducted following the ‘Atacama soil microbiome tutorial’ by Qiime2docs along with customized programme scripts (https://docs.qiime2.org/2019.1/). Briefly, the raw data FASTQ files were imported into a format compatible with the QIIME2 system using the qiime tools import programme. Demultiplexed sequences from each sample were quality filtered, trimmed, denoised and merged, and then, the chimeric sequences were identified and removed using the QIIME2-Dada2 plugin to obtain the feature table of amplicon sequence variants (ASVs). The QIIME2 feature-classifier plugin was used to align ASV sequences to a pre-trained GREENGENES 13_8 99% database (trimmed to the V3–V4 region bound by the 338F/806R primer pair) and generate the taxonomy table. Contaminating mitochondrial and chloroplast sequences were filtered using the QIIME2 feature-table plugin. Various statistical methods, including ANCOM, ANOVA, Kruskal–Wallis, linear discriminant analysis effect size (LEfSe) and DESeq2, were employed to identify bacteria with different abundances among samples and groups. Diversity metrics were calculated using the core-diversity plugin within QIIME2. Alpha diversity indices at the feature level, such as observed OTUs (Operational Taxonomic Units), the Chao1 richness estimator, the Shannon diversity index and Faith’s phylogenetic diversity index, were calculated to estimate the microbial diversity within each sample. Beta diversity distance measurements, including Bray–Curtis, unweighted UniFrac and weighted UniFrac, were performed to investigate the structural variation of microbial communities across samples, and the results were visualized using principal coordinate analysis (PCoA) and nonmetric multidimensional scaling. Partial least squares discriminant analysis was also used as a supervised model to reveal the variation of microbiota among groups, employing the ‘plsda’ function in the R package ‘mixOmics’. Redundancy analysis was performed to explore the association of microbial communities with environmental factors based on the relative abundance of microbial species at different taxonomic levels using the R package ‘vegan’. Co-occurrence analysis was performed by calculating Spearman’s rank correlations between dominant taxa, and the network diagram was used to display the associations among taxa. Additionally, the potential functional profiles of microbial communities based on KEGG Orthologs were predicted using PICRUSt. Unless otherwise stated, standard parameters were used in the analysis.

### Statistical analysis

For comparisons of two groups, significance was tested by an unpaired two-tailed Student’s t-test after assessing normality using the Shapiro–Wilk test. For more than two groups, we first checked the homogeneity of variances using Levene’s test. If variances were equal, we used a one-way ANOVA followed by a Tukey test with an adjusted *P*-value for multiple comparisons. If variances were unequal, Welch’s ANOVA was employed instead. For non-normally distributed data, two groups were compared using the Wilcoxon–Mann–Whitney test, and for more than two groups, the Kruskal–Wallis test was used with the Dunn–Bonferroni test (GraphPad Prism 8). The data between experimental groups were considered significant as *P<0.05. Effect sizes (Cohen’s d and η²) were calculated to provide context to the magnitude of observed effects.

## Results

### Alterations in the gut microbiota following FMT and ozonated water enema

**Fig. 1.**
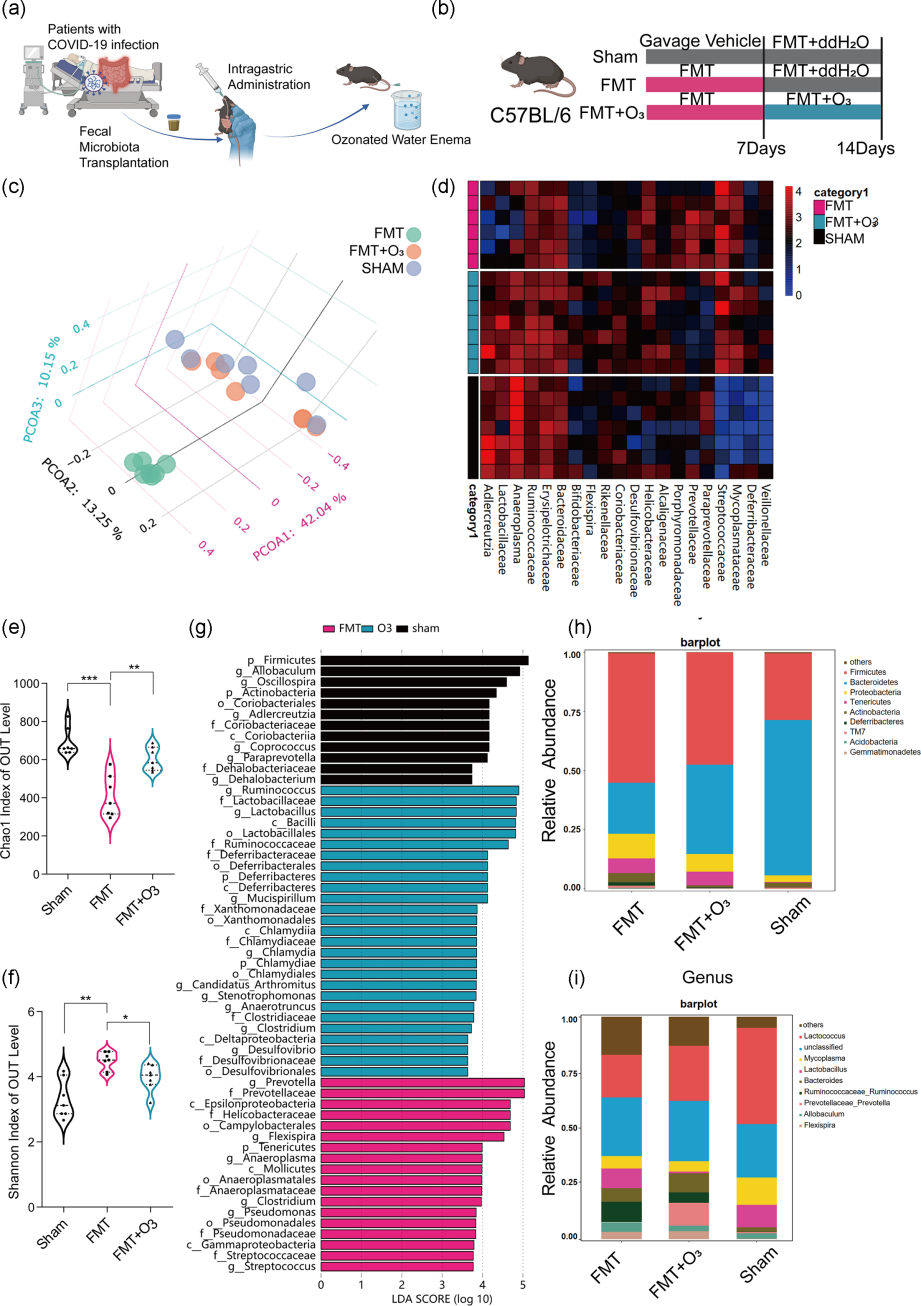
FMT from COVID-19 patients alters the gut microbiota of mice. (**a**) Collection of faeces from COVID-19-infected patients, modelling of mice by FMT and ozone water enema intervention treatment experiment. (**b**) Experimental setup: WT mice were randomly assigned to three groups: the Sham group received no intervention for 14 days, while the FMT and FMT+O_3_ groups underwent FMT from COVID-19 patients for the initial 7 days. Additionally, the FMT+O_3_ group received ozonated water enemas for the subsequent 7 days. The experiment was analysed after a total duration of 14 days. (**c**) PCoA based on weighted UniFrac distances of the relative abundances of bacterial OTUs after FMT and ozonated water enemas. *n*=7/group. (**d**) Hierarchical clustering and a heatmap were utilized to illustrate the relative abundance of taxa summarized at the genus level across different groups of interest for the top 20 OTUs. The colour gradient on the heatmap represents the relative abundance of each taxon within each sample, with red indicating the highest abundance and green indicating the lowest (**e, f**). Chao1 and Shannon indices were used as indicators of community richness and diversity. *n*=7/group. (**g**) Analysis of intestinal microbiota at the phylum level. (**h**) Analysis of intestinal microbiota at the gene level. (**i**) Linear discriminant analysis scores for different taxa abundances. Data are mean±sem, with *P<0.05, **P<0.01 and ***P<0.001 for significance.

In an effort to understand the impact of SARS-CoV-2 on the gut microbiota, we conducted a study involving the transplantation of faecal flora from COVID-19 patients to mice. A total of 21 COVID-19 patients were recruited from the Respiratory Critical Care Emergency Department of Shanghai Oriental Hospital. The criteria for inclusion and exclusion of faecal sample collection are outlined in Table S1. To assess the effects of FMT on the intestinal microbiota of mice, faecal samples were collected from three groups of eight mice each, following a 7-day intervention with ozone water enemas (Fig. 1a, b). The samples were then subjected to 16S rRNA gene sequencing. The PCoA revealed a significant shift in the intestinal microbial profile as a result of FMT (Fig. 1c). A heatmap analysis of the relative abundance at the family level showed that the FMT group had higher levels of *Mycoplasmataceae*, *Desulfovibrionaceae*, *Streptococcaceae* and *Prevotellaceae* compared to the Sham group (Fig. 1d). Notably, the FMT group exhibited lower abundance of *Adlercreutzia*, *Lactobacillaceae*, *Bacteroidaceae* and *Anaeroplasma* compared to the Sham group (Fig. 1d). In contrast, the FMT+O_3_ group showed higher proportions of *Adlercreutzia*, *Lactobacillaceae* and *Anaeroplasma* compared to the Sham group. Interestingly, the FMT group displayed a reduction in alpha diversity, suggesting that the introduction of COVID-19 patients' gut microbiota into mice leads to the depletion of specific bacterial taxa. Further evaluation in the FMT+O_3_ group revealed an increase in both the Chao1 and Shannon indices, although these remained lower than those observed in the Sham group (Fig. 1e, f). This finding suggests that ozone water enemas may potentially enhance the abundance of bacterial microbiota in mice. At the phylum level, the FMT group showed an increased relative abundance of *Firmicutes*, *Tenericutes*, *Actinobacteria* and *Deferribacteres* compared to the Sham group. Conversely, the presence of *Bacteroidota* was reduced in the FMT group. Additionally, the ozonated water enema treatment group exhibited an increased relative abundance of the genera *Prevotellaceae_Prevotella* and *Bacteroides*, while the abundance of *Mycoplasma* and *Lactobacillus* decreased (Fig. 1g, h). The three main groups of changing flora and their biological significance are shown in Table 2. To further explore the differences in the gut microbiota among the groups, we conducted a LEfSe analysis. This analysis revealed significant differences in the relative abundance of gut microbiomes among the three groups (Fig. 1i). These findings underscore the potential of ozone water enemas as a therapeutic intervention to modulate the gut microbiota in mice, which could have implications for the management of systemic symptoms in COVID-19 patients.

**Table 2.** Bacterial composition across groups

Bacterial family/genus	Sham	FMT	FMT+O₃	Significance/clinical interpretation
***Mycoplasmataceae***	↓	↑	↓	A major pathogen causing respiratory diseases, ranging from mild pharyngitis to severe pneumonia [103,104]
***Desulfovibrionaceae***	↓	↑	–	Sulphate reduction, associated with intestinal flora dysbiosis and intestinal inflammation [105]
***Streptococcaceae***	↓	↑	–	Opportunistic pathogens, can drive inflammation, affect host health through multiple mechanisms [106]
***Prevotellaceae***	↓	↑	↑	Excessive amounts may cause inflammation, triggering an excessive inflammatory response leading to tissue damage and disease progression [107]
***Adlercreutzia***	↑	↓	↑	Beneficial, associated with the production of SCFA, anti-inflammatory properties, able to inhibit inflammatory responses *in vitro* and *in vivo* [108]
***Lactobacillaceae***	↑	↓	↑	Maintains gut microecological balance, participates in substance metabolism, regulates immunity and enhances intestinal barrier function [109]
***Bacteroidaceae***	↑	↓	↑	Maintains gut microecological balance, participates in substance metabolism, regulates immunity and enhances intestinal barrier function [110]
***Anaeroplasma***	↑	↓	↑	Maintains intestinal microecology stability, enhancing immune defence and pathogen resistance [111]

### Alteration in intestinal microbiota affects the function of the intestinal epithelial barrier

**Fig. 2.**
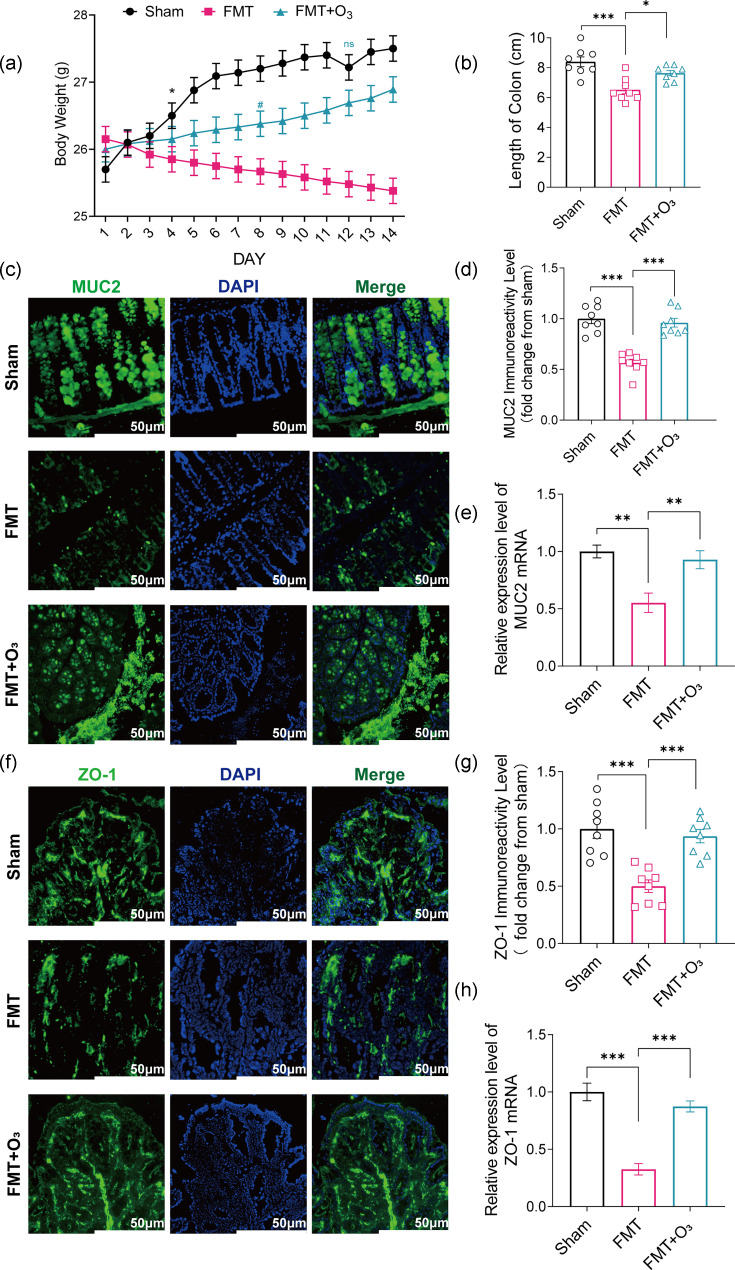
Altered intestinal epithelial barrier function following FMT. (**a**) Trends in body weight changes over 14 days in three groups of mice (g). (**b**) Statistics of intestinal length in three groups of mice at 14-day sampling (*P=0.0414, ^#^*P*=0.0228). (**c**) Immunostaining of MUC2 (green) and DAPI (blue) showing in three groups of colon tissues. Scale bars represent 50 µm. (**d**) Representative immunofluorescence staining level for MUC2, fold change versus the Sham group. (**e**) Detection of factor colon tissue levels of MUC2 using real-time PCR. (**f**) Immunostaining of ZO-1 (green) and DAPI (blue) showing in three groups of colon tissues. Scale bars represent 50 µm. (**g**) Representative immunofluorescence staining level for ZO-1, fold change versus the Sham group. (**h**) Detection of factor colon tissue levels of ZO-1 using real-time PCR. *n*=8 in each group. Data are mean±sem, with *P<0.05, **P<0.01 and ***P<0.001 for significance.

The primary objective of our study was to investigate the impact of microbiota associated with COVID-19 patients on the outcomes of WT mice following FMT and to conduct a comprehensive analysis of gut phenotypes. Our observations revealed that the mice experienced weight loss after undergoing FMT, a condition that improved with subsequent ozone water enema treatment (Fig. 2a). On day 4, a statistically significant difference in mean body weight was observed between the Sham and FMT groups (*P*=0.0414). By day 8, a significant difference in mean body weight was also noted between the FMT and FMT+O_3_ groups (*P*=0.0228). However, on day 12, there was no statistically significant difference in body weight between the Sham and FMT+O_3_ groups. This indicates that ozonated water treatment enhanced the body functions of the mice and reversed the weight loss caused by FMT. Furthermore, we found that the intestinal length of mice in the FMT group was shorter during the sampling period, suggesting that the transplantation of faecal microbiota from COVID-19 patients may lead to decreased appetite, compromised immunity and intestinal atrophy in the mice (Fig. 2b). Experimental data from Abdul Rashid *et al*. highlight the critical role of the gut microbiota in maintaining the integrity of the intestinal barrier, yet its specific impact on COVID-19 patients remains to be elucidated. Severe COVID-19 often results in multi-organ failure, which can severely impact the prognosis of the disease [42,43]. To evaluate the effects of SARS-CoV-2 on the intestine, we examined the intestinal epithelium of mice after cecum transplantation from COVID-19 patients. Immunofluorescence staining of tissue sections showed a significant decrease in Muc2 protein content in the ileal epithelium of mice in the FMT group compared to the Sham group, as well as a significant decrease in the content of the tight junction protein, ZO-1 (Fig. 2c, d, f, g). Additionally, we measured the mRNA content of Muc2 and ZO-1 in the colon tissues of the three groups using real-time PCR (RT-PCR), and the results showed a significant decrease in the FMT group compared to both the Sham and FMT+O_3_ groups (Fig. 2e, h). These findings suggest that the gut microbiota of COVID-19 patients adversely affects the intestinal health of mice, leading to disrupted intestinal barrier function and impaired mucus barrier, which may contribute to systemic complications. The improvements observed with ozone water enema treatment indicate its potential as a therapeutic intervention to mitigate these effects.

### Ozone water enema improves intestinal inflammation induced by FMT

**Fig. 3.**
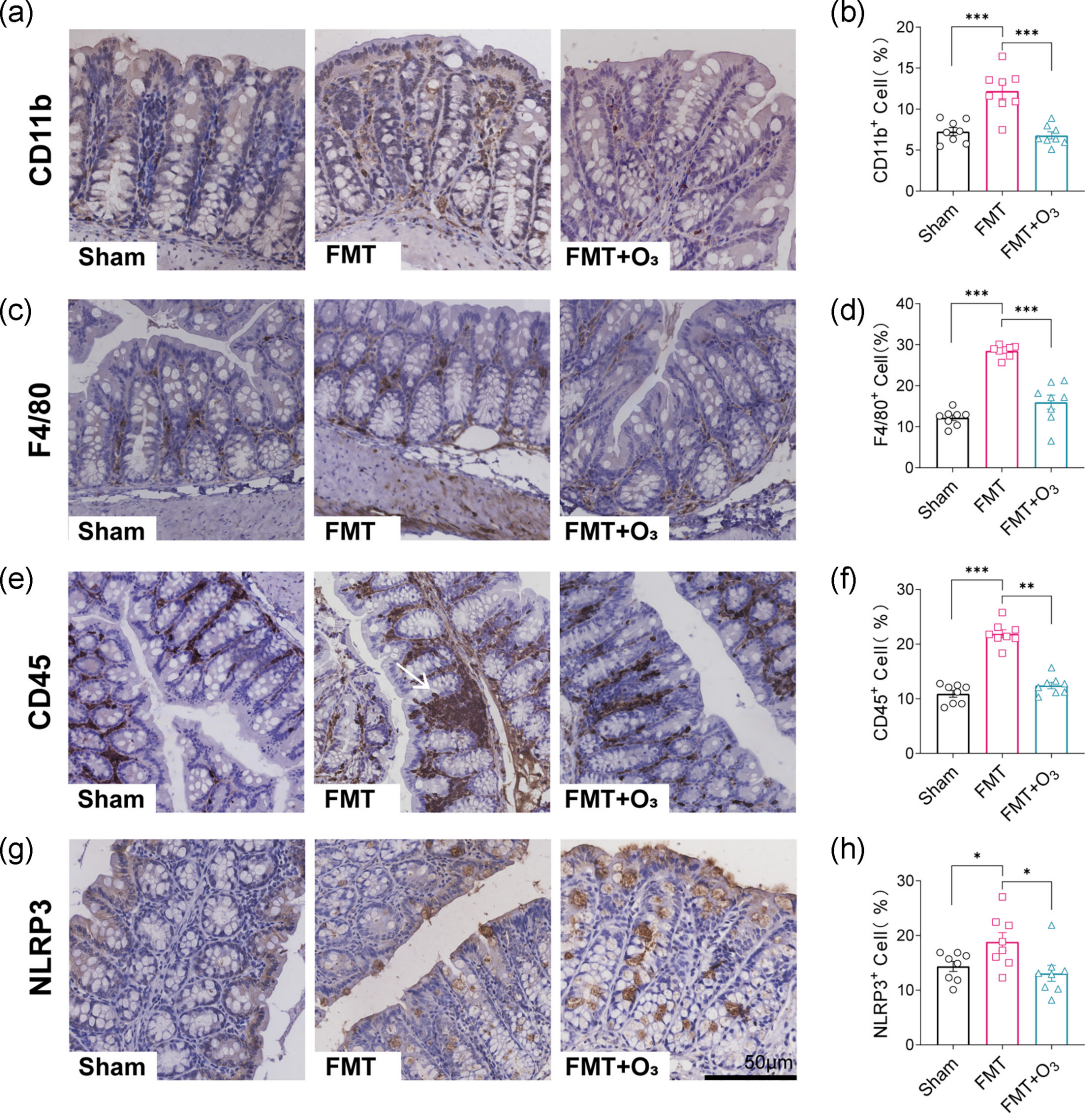
Intestinal inflammatory alterations after FMT and ozone water enema treatment. (**a, c, e, g**) Representative pictures of IHC staining of colon sections CD11b, F4/80, CD45 and NLRP3 of Sham, FMT and FMT+O_3_; giant pannus nodules (white arrow); scale bar: 50 µm. (**b**) Histological quantification of CD11b-positive cells per view field. (**d**) Histological quantification of F4/80-positive cells per view field. (**f**) Histological quantification of CD45-positive cells per view field. (**h**) Histological quantification of NLRP3-positive cells per view field. This experiment was repeated independently three times, and similar results were obtained. *n*=8 in each group. Data are mean±sem, with *P<0.05, **P<0.01 and ***P<0.001 for significance.

After undergoing FMT, mice demonstrated a markedly intensified inflammatory response, with the recruitment of inflammatory monocytes playing a pivotal role in intestinal injury. Immunohistochemical (IHC) staining for CD11b and F4/80 revealed a significant twofold increase in F4/80 cell infiltration in the colon of mice post-FMT compared to the Sham group (Fig. 3a, c). Moreover, IHC staining for CD45 indicated elevated leucocyte infiltration in the gut of mice, forming dense clusters (Fig. 3e). Compared to the Sham group, FMT mice exhibited significantly heightened inflammatory infiltration in the intestine, characterized by shorter villi, apical rupture and infiltration of inflammatory cells in the muscular propria, along with the presence of giant pannus nodules (Fig. 3e). Given the observed increase in leucocyte infiltration in the FMT group of mice, our subsequent focus was directed towards investigating gut changes to elucidate the functional mechanisms underlying the amplified systemic inflammatory response in these mice. However, following the notable reduction in macrophage and leucocyte infiltration in the FMT+O_3_ group of mice compared to the FMT group, along with a tendency towards lower CD11b, F4/80 and CD45 expression (Fig. 3b, d, f), our focus shifted to understanding the mechanisms by which ozonated water enemas exert anti-inflammatory effects, leading to the resolution of inflammation in the FMT group. This suggests that ozonated water enemas may help mitigate the heightened systemic inflammatory response observed in FMT mice by modulating intestinal inflammation and immune cell infiltration. After IHC staining for the NLRP3 inflammatory factor, we observed an intriguing result. There was a significant increase in NLRP3 expression in colon tissue sections in the FMT group compared to the Sham group (Fig. 3g). Additionally, there was a significant tendency towards decreased NLRP3 expression in colon tissue after administration of ozone water enema treatment (Fig. 3h). These findings suggest that the gut microbiota from COVID-19 patients can trigger a robust inflammatory response in mice, potentially contributing to intestinal injury. The reduction in inflammatory markers and NLRP3 expression following ozone water enema treatment indicates its potential as a therapeutic intervention to mitigate these inflammatory effects.

### Changes in gut microbiota induce systemic inflammation

**Fig. 4.**
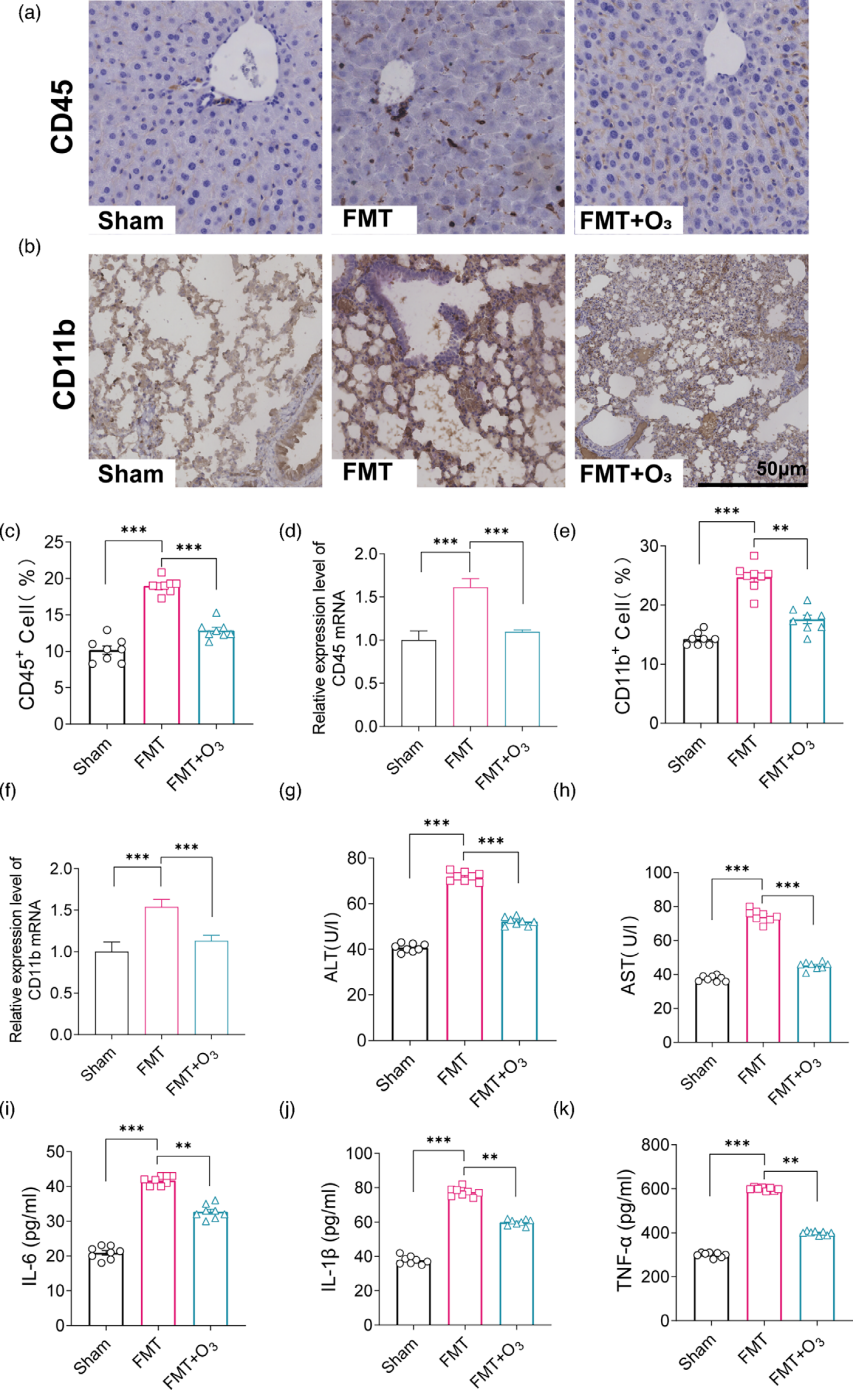
Ozone water enema attenuates lung and liver injury induced by FMT. (**a, c**) Representative images of CD45 IHC staining of Sham, FMT and FMT+O_3_ liver sections and quantitative analysis of positive expression; scale bar: 50 µm. (**b, e**) Representative images of CD11b IHC staining of Sham, FMT and FMT+O_3_ lung sections and quantitative analysis of positive expression; scale bar: 50 µm. (**d**) Levels of CD45 in factor liver tissue using RT-PCR expression quantitative analysis; scale bar: 50 µm; *n*=5 in each group. (**d**) Levels of CD45 in factor liver tissues were detected using RT-PCR. (**f**) RT-PCR was used to detect CD11b levels in factor lung tissue. (**g**) Serum levels of ALT (U/l) in Sham, FMT and FMT+O_3_ mice. (**h**) AST levels in serum of Sham, FMT and FMT+O_3_ groups of mice (U/l). (**i–k**) Serum levels of IL-6, IL-1β and TNF-α in Sham, FMT and FMT+O_3_ groups of mice (pg/ml). *n*=8 in each group. Data are mean±sem, with *P<0.05, **P<0.01 and ***P<0.001 for significance.

To further evaluate the overall health of mice following FMT from critically ill COVID-19 patients, we conducted a comprehensive examination of the liver, lungs and blood across three groups of mice. IHC staining for CD45 revealed an increase in leucocyte infiltration in the liver post-FMT, accompanied by enhanced recruitment of inflammatory monocytes, which contribute to liver damage (Fig. 4a, c). In the preceding conclusion, we have already demonstrated that FMT-induced gut microbiota dysbiosis and gut epithelial barrier disruption lead to an inflammatory response. Similar inflammatory responses were observed in the lungs, as evidenced by IHC staining of lung tissue sections for CD11b (Fig. 4b, e). On day 14 of the experiment, mice that received FMT from critically ill COVID-19 patients exhibited severe pulmonary fibrosis and increased intra-alveolar exudation. RT-PCR analysis of liver and lung tissues collected on day 14 revealed significantly higher mRNA levels of CD45 in liver tissues and CD11b in lung tissues in the FMT group compared to the Sham group (Fig. 4d, f). Ozone water enema intervention reversed this inflammatory trend in both organs when compared to the FMT group. Haematological examination of blood samples collected on day 14 showed a significant increase in AST and ALT levels in FMT mice, indicating liver inflammation (Fig. 4g, h). Additionally, the expression of pro-inflammatory cytokines IL-6, IL-1β and TNF-α was significantly upregulated in the FMT group compared to the Sham group and decreased following ozone water enema treatment (Fig. 4i–k). These results suggest that FMT from critically ill COVID-19 patients can induce systemic inflammation affecting multiple organs, including the liver and lungs. The increase in liver enzymes and pro-inflammatory cytokines indicates liver and systemic inflammation. Ozone water enema treatment appears to mitigate these inflammatory effects, suggesting its potential as a therapeutic intervention to improve overall health post-FMT.

### Exploration of the mechanism of ozone water enema to ameliorate FMT-induced intestinal inflammation

**Fig. 5.**
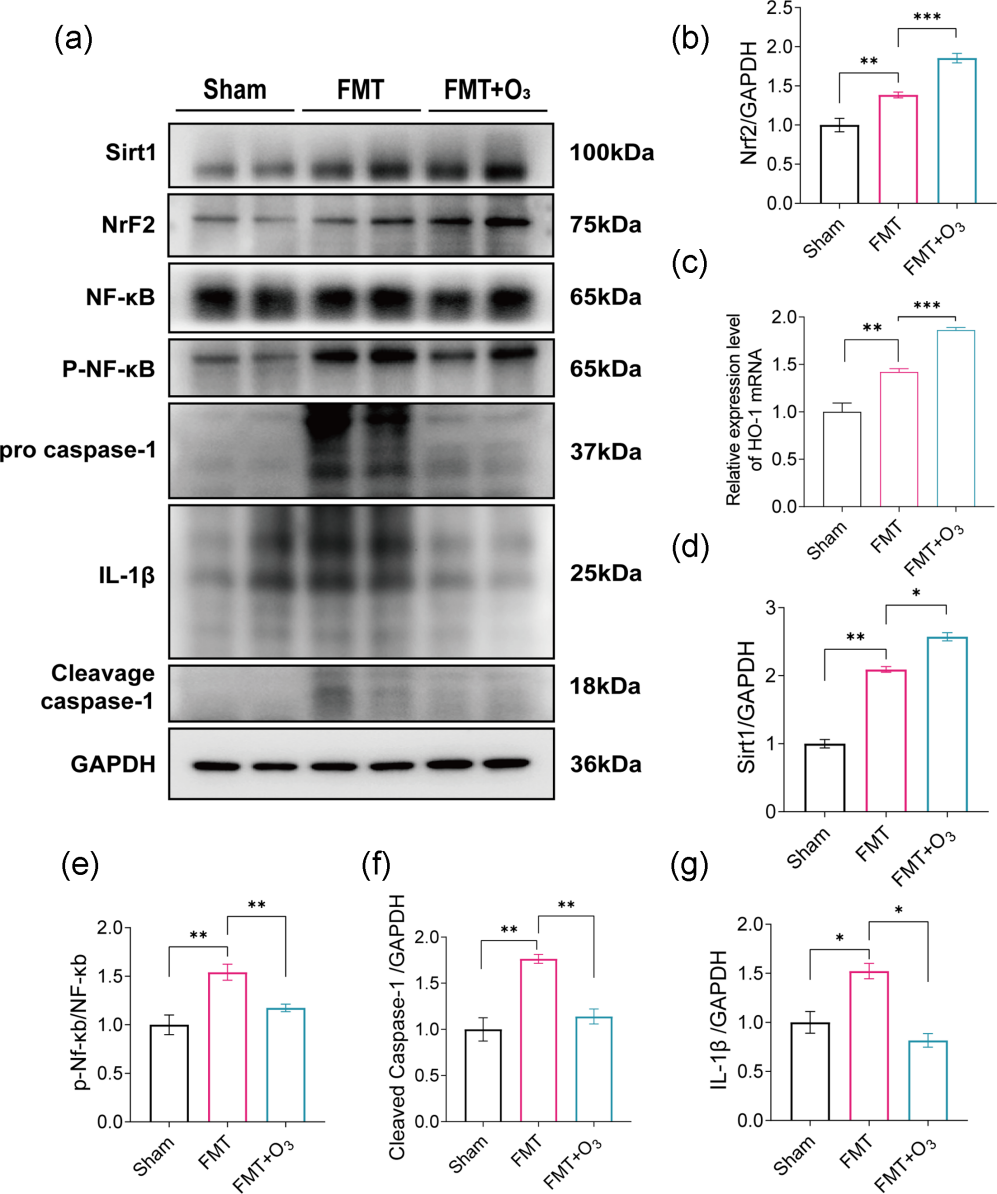
Ozone water enema treatment inhibits IL-1β and activates Nrf2 oxidative stress. (**a**) Western blotting bands of SIRT1, NF-κB, Nrf2, caspase-1, IL-1β and cleaved caspase-1 in colon tissue. (**b, d–g**) Analysis of SIRT1, NF-κB, Nrf2, cleaved caspase-1 and IL-1β in colon tissue. (**c**) Detection of factor colon tissue levels of HO-1 using RT-PCR. Data are mean±sem, with *P<0.05, **P<0.01 and ***P<0.001 for significance.

Previous experiments have showed that transplanting faecal microbiota from critically ill COVID-19 patients into mice alters the gut microbial community, potentially reducing microbiota numbers and triggering a significant systemic inflammatory response. In terms of protein expression in colonic tissues, levels of SIRT1, Nrf2, NF-κB, caspase-1 and IL-1β were measured. The protein levels of SIRT1 and Nrf2 were found to be higher in the FMT group compared to the Sham group, and these levels continued to increase even after treatment with ozone water enemas (Fig. 5a, b, d). In both the FMT group and the FMT+O_3_ group, protein levels in colonic tissues showed a decrease in the expression of NF-κB, cleaved caspase-1 and IL-1β (Fig. 5a, e–g). Numerous studies have indicated that repeated enemas with low-dose ozonated water can activate the Nrf2 pathway. This activation leads to an increase in the expression of various antioxidant enzymes, such as glutathione peroxidase and catalase, which can help reduce tissue damage and organ dysfunction caused by oxidative stress [44,45]. In the FMT+O_3_ group, the expression of HO-1 mRNA in the intestinal tissues of mice was significantly higher compared to the FMT group (Fig. 5c). At the protein level in colonic tissues, the expression of HO-1 was also significantly increased in the FMT+O_3_ group compared to the FMT group. These findings suggest that ozonated water enemas may enhance the expression of antioxidant enzymes and reduce inflammation, potentially mitigating the adverse effects of COVID-19-associated microbiota on the gut and systemic health.

## Discussion

The COVID-19 pandemic has profoundly impacted epidemiology and public health [46]. Its high transmissibility has caused widespread disruption and influenced the epidemiology of other infectious diseases [47]. Moreover, it has affected mental health and the outcomes of non-communicable diseases [48,49]. Emerging evidence shows that COVID-19 can impact the gastrointestinal tract beyond the respiratory system [50,51]. Severe COVID-19 patients often exhibit gut dysbiosis, characterized by reduced microbial diversity and increased opportunistic pathogens [52,53]. Severe COVID-19 patients often exhibit gut dysbiosis, characterized by reduced microbial diversity and increased opportunistic pathogens [54,56]. Viral particles and the ACE2 receptor, the virus’s entry point, have been found in gastrointestinal tract cells, indicating potential direct infection and replication [11,57,59]. The gut microbiota, crucial for gut health and immunity, is also affected by COVID-19 [60], with alterations linked to heightened systemic inflammation and immune dysregulation [61].

The long-term epidemiological impact of COVID-19 remains an active research area. In this context, ozonated water enemas present a promising therapeutic approach due to their potential to modulate the gut microbiota and mitigate inflammation. Recent clinical trials and case reports by Rapone *et al.* have begun to explore the efficacy of ozone therapy for a variety of conditions, including its antibacterial and anti-inflammatory properties [62,64]. Ozonated water enemas show promise as a therapeutic approach for modulating the gut microbiota and reducing inflammation, though specific studies on their use in COVID-19 patients are limited [65].

The treatment outcomes of ozonated water enemas may vary between mild and severe COVID-19 patients. For mild cases, they could serve as a preventive measure to maintain a healthy gut microbiota [52]. In contrast, for severe cases, they might need to be combined with other therapies to achieve a more significant impact on the gut microbiota and immune regulation [61].

In our study, mice transplanted with intestinal faecal microbiota from critically ill COVID-19 patients exhibited impaired intestinal epithelial barriers, disrupted integrity, reduced ZO-1 expression and decreased Muc2 levels. There was also a significant increase in intestinal inflammatory infiltration and elevated expression of inflammatory factors such as CD11b, F4/80, CD45 and NLRP3 in colonic tissues. We hypothesized that the compromised intestinal barrier function post-SARS-CoV-2 infection exacerbates intestinal inflammatory cell infiltration and cytokine release. Additionally, liver, lung and systemic inflammation were observed, and hepatic CD45 and lung F4/80 expression were significantly higher in mice receiving FMT compared to the Sham group. Elevated AST and ALT levels and increased pro-inflammatory cytokines such as IL-1β, IL-6 and TNF-α were also evident in the FMT group. Overall, our study highlights the extensive impact of SARS-CoV-2 infection on various organs and emphasizes the role of impaired intestinal barrier function in promoting inflammation.

Ozonated water enema therapy is believed to exert several potential therapeutic effects. The introduction of ozonated water into the colon may promote oxygen release, enhancing cellular metabolism and tissue oxygenation and supporting the growth of beneficial anaerobic bacteria while inhibiting harmful ones [66,68]. Additionally, ozone’s oxidative properties may confer antimicrobial and antifungal effects, aiding in eliminating pathogenic micro-organisms [66,69]. Furthermore, low-dose ozone exposure may activate the antioxidant response, stimulating antioxidant enzyme expression and modulating redox balance in the intestinal tissues, thus mitigating oxidative stress-induced damage and promoting tissue repair [45,70]. Previous studies have demonstrated significant anti-inflammatory effects of ozone water enemas in various inflammatory conditions. However, previous studies have demonstrated significant anti-inflammatory effects of ozone water enemas in various inflammatory conditions, including colitis, pneumonia and viral infections [18,71]. Ozone therapy shows promise in modulating the gut microbiota and reducing inflammation, particularly in COVID-19 and related complications [72]. While our study and previous research highlight its therapeutic potential, it is crucial to critically assess its oxidative properties and potential hazards [73]. Ozone (O₃), a strong oxidizing agent, generates ROS upon interaction with biological molecules [74]. Moderate ROS levels can enhance cellular metabolism and pathogen clearance, but excessive ROS lead to oxidative stress, damaging cellular components and contributing to diseases like inflammation and degeneration [75,76]. The potential side effects of ozone therapy are primarily related to its oxidative properties. These include local tissue damage, such as irritation and damage to the gastrointestinal mucosa (causing symptoms like abdominal pain, bloating and diarrhoea), and the exacerbation of existing inflammatory conditions or the development of new ones due to systemic oxidative stress [77,78]. Despite these potential risks, the safety profile of ozone therapy is generally favourable when administered under controlled conditions, as indicated by current literature and our study. We conducted rigorous safety monitoring, including pre-treatment screening to identify conditions that might increase adverse reaction risks, careful control of ozone concentration and treatment duration to minimize oxidative stress and post-treatment monitoring to detect any immediate or delayed adverse reactions.

Further research is needed to fully understand the long-term effects of ozone therapy and optimize treatment protocols to minimize potential risks. In conclusion, ozone therapy shows significant therapeutic potential for modulating the gut microbiota and reducing inflammation, particularly in COVID-19 and related complications. However, it is essential to acknowledge and carefully manage its oxidative properties and potential hazards. Our study observed that ozone water enema intervention led to improvements in the gut flora of mice, with a rebound in the reduced abundance of beneficial flora and a decrease in harmful bacteria. This indicates that ozone treatment plays a crucial role in restoring the balance of intestinal micro-organisms. Furthermore, it enhanced the integrity of the intestinal barrier and increased the expression of mucin, evident from the elevated levels of colon epithelial ZO-1 and MUC2 expression [66,67]. Previous studies have demonstrated ozone’s ability to modulate the activity of immune cells and regulate cytokine production within the intestinal mucosa. This immunomodulatory effect may play a role in reducing the inflammatory response within the gut [69,79]. Additionally, intervention with ozone water enemas has been associated with decreased expression of inflammatory cells and inflammatory factors in the liver, lungs and blood. Overall, these findings suggest that ozone water enema therapy exerts immunomodulatory effects, potentially contributing to the regulation of the inflammatory response.

**Fig. 6.**
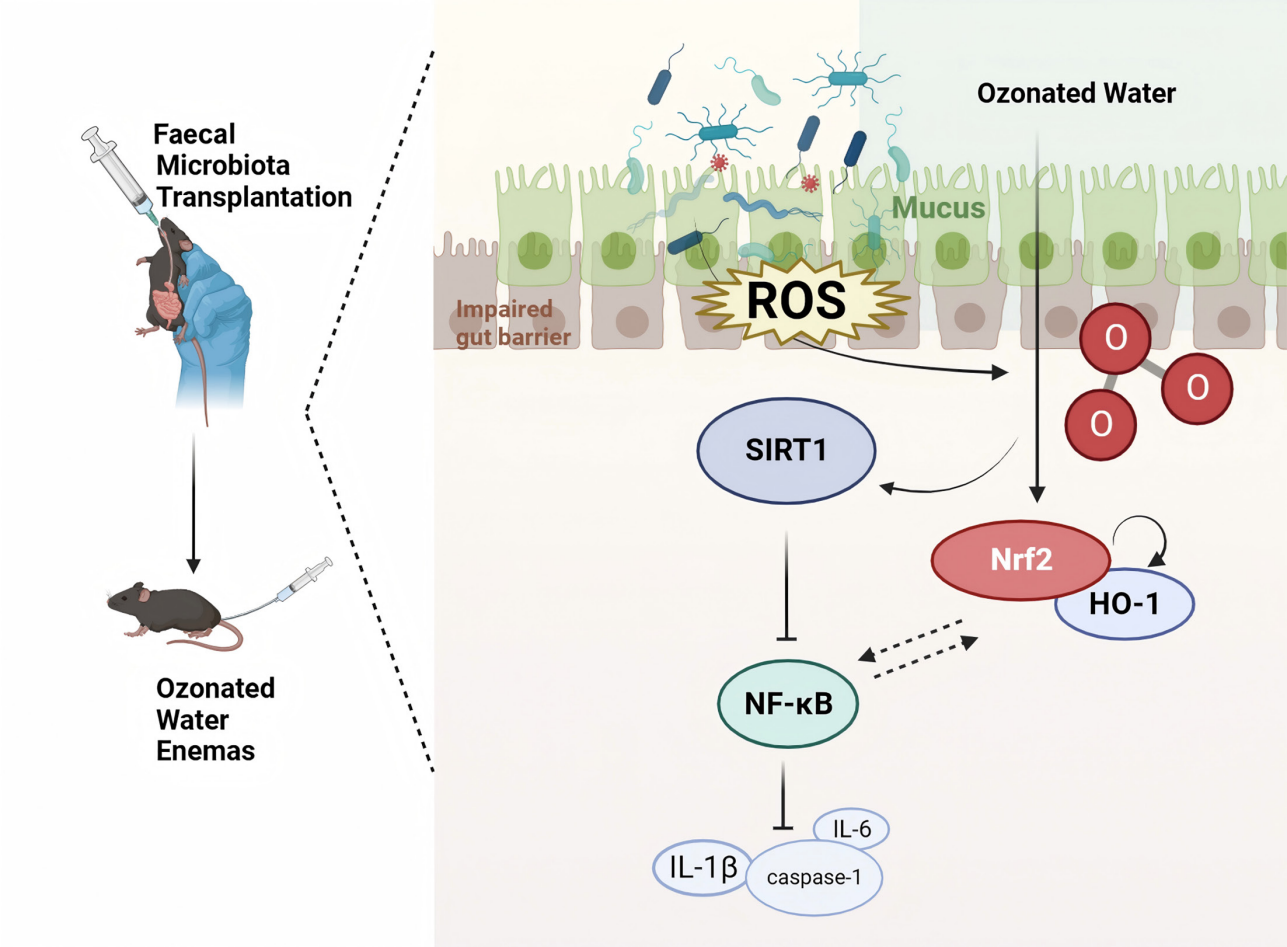
Ozone water enema has been shown to improve intestinal and systemic symptoms in mice receiving FMT from COVID-19 patients. This improvement is attributed to the activation of the Nrf2-HO-1 signalling pathway and overexpression of SIRT1, which enhances the body’s ability to resist oxidative stress. Additionally, ozone water enema inhibits inflammatory factors such as NF-κB and its downstream IL-1β and caspase-1.

Oxidative stress, often resulting from the overproduction of ROS, is widely recognized as a pivotal factor in cellular damage [80,81]. Nrf2 is a vital protective transcription factor involved in the body’s response to oxidative stress and cellular defence [82]. Our study observed the activation of Nrf2 at the protein level in the FMT and FMT+O_3_ groups, alongside an increase in the mRNA content of HO-1 in colonic tissues. It is well known that HO-1 mRNA and protein expression are upregulated in response to oxidative stress and cellular injury, and Nrf2 can directly regulate HO-1 promoter activity [83,84]. After the transplantation of faecal flora from critically ill COVID-19 patients, mice developed an inflammatory response in the intestines, stimulating oxidative stress. Our study revealed that ozonated water enemas increased faecal microbiota abundance in mice, effectively reducing damage to the intestinal barrier and inflammation. Furthermore, treatment with ozonated water effectively activated the intestinal Nrf2-HO-1 pathway and enhanced its oxidative stress capacity. SIRT1 is a conserved, NAD^+^-dependent class III histone deacetylase [85,86]. It has been reported that inhibition of SIRT1 reduced the interaction between SIRT1 and Nrf2, consequently inhibiting the activation of the Nrf2 pathway and leading to the down-regulation of downstream antioxidant genes [87]. Activation of SIRT1 and Nrf2 has been shown to play an important role in a variety of diseases [88,89]. In our study, we observed activation of intestinal SIRT1 in mice that received FMT, and its expression levels continued to increase following ozone water enema treatment. Based on these findings, we propose that the activation of SIRT1-Nrf2 crosstalk interaction enhances the body’s capacity to counter oxidative stress after ozone water enema treatment. Recent studies have revealed that SIRT1 can reduce the inflammatory response by inhibiting multiple pathways involved in inflammation [90,93]. SIRT1 has been discovered to decrease the release of inflammatory mediators by inhibiting the activation of NF-κB, thereby mitigating the inflammatory response [94,95]. The overexpression of SIRT1 has been shown to decrease the transcriptional activity of NF-κB protein [96,97] (Fig. 6).

The potential of ozone therapy for disease prevention is being explored due to its antioxidant and immunomodulatory properties. Research indicates that ozone can stimulate the body’s antioxidant enzyme system, prompting the production of superoxide dismutase, glutathione peroxidase and other free radical scavengers, thereby enhancing the body’s antioxidant capacity. This mechanism may aid in preventing conditions such as ageing, tumourigenesis and cerebrovascular accidents. For instance, by providing ample oxygen to tumour sites, ozone can selectively inhibit tumour growth and activate the immune system, leading to an increase in tumour necrosis factor and thereby bolstering the body’s resistance to infections and tumours [98]. Ozone therapy is thought to invigorate the immune system through the activation of immune cells like macrophages and natural killer cells, thus augmenting the body’s defence mechanisms. This activation results in a non-specific desensitizing effect and promotes the phagocytic activity of white blood cells, thereby strengthening the body’s immune defenses [99]. Moreover, ozone induces the expression of antioxidant enzymes, which not only assist in scavenging ROS but also support the overall function of the immune system. Studies by Jeyaraman *et al*. have shown that ozone therapy can improve metabolic function by enhancing the metabolism of red blood cells and increasing the oxygen-carrying capacity of haemoglobin. This improvement in cellular respiration and energy production is crucial for maintaining overall metabolic health [62]. In our study, we confirmed this concept by examining intestinal protein levels in mice after receiving faecal flora transplantation from critically ill COVID-19 patients. We observed a significant up-regulation of SIRT1 and NF-κB in the FMT group compared to the Sham group. Furthermore, the expression of SIRT1 continued to increase, while NF-κB expression decreased following ozone water enema treatment, despite the presence of persistent inflammatory effects. In comparison to the FMT group, the expression of IL-1β and cleaved caspase-1 tended to decrease in the FMT+O_3_ group. These findings suggest that SIRT1, through the inhibition of NF-κB activation, reduces the secretion of inflammatory mediators such as IL-1β and cleaved caspase-1, ultimately leading to a reduction in the inflammatory response after ozone water enema treatment. Zheng *et al*. demonstrated that the Nrf2-NF-κB signalling pathway can be modulated by activating SIRT1, leading to a neuroprotective effect on the organism [100]. Additionally, it has been shown that the up-regulation of antioxidant transcription factors SIRT1 and Nrf2, coupled with the down-regulation of the pro-inflammatory factor NF-κB, can exert a protective effect on the liver [101]. Based on our findings, we propose a hypothesis suggesting that the anti-oxidative stress and anti-inflammatory properties of ozone primarily occur through the activation of the Nrf2-HO-1 signalling pathway and the up-regulation of SIRT1, leading to sustained enhancement of Nrf2-mediated anti-oxidative stress. Additionally, SIRT1 inhibits NF-κB, thereby reducing the expression of inflammatory factors such as caspases and IL-1β.

Furthermore, ozonated water enemas leverage the strong oxidizing properties of ozone to eliminate pathogens, making them a treatment option for conditions like ulcerative colitis. However, there is a paucity of in-depth research regarding the role of ozone water therapy (including enemas) in disease prevention, enhancement of autoimmunity, elevation of metabolic levels and augmentation of beneficial intestinal flora in healthy individuals or mice [102]. In our experimental design, we did not administer ozonated water alone as an intervention. This absence might influence the positive control aspect of the treatment. Looking ahead, more experiments are warranted. These should include comparing the effects of ozonated water enemas as a preventive measure against COVID-19 with a Sham group and ozonated water enema alone. This approach will help determine the preventive potential of ozonated water enemas and their impact on gut microbiota and systemic health. While our current study focuses on the anti-inflammatory and oxidative stress effects of ozone water in the intestinal tract, further exploration is necessary to understand its potential in treating COVID-19 microbiota disorders. This includes investigating its effects on other COVID-19 complications and sequelae, such as respiratory ARDS, respiratory failure, cardiovascular myocarditis, cardiac arrhythmia, renal injury and transient or long-term cognitive dysfunction. Future research should prioritize well-designed, multicentre, randomized controlled trials to rigorously evaluate the efficacy and safety of ozonated water enemas in COVID-19 patients [52]. These trials should aim to determine the optimal dosage and treatment duration, considering the varying degrees of gut dysbiosis and the unique clinical profiles of different patient groups [52]. Additionally, long-term follow-up studies are essential to assess the sustained effects of ozonated water enemas on gut microbiota composition and overall health outcomes, including the potential for preventing post-acute sequelae of COVID-19.

By addressing these research gaps, the scientific community can better understand the therapeutic potential of ozonated water enemas and pave the way for their integration into clinical practice for managing COVID-19-related gut dysbiosis. We suggest several avenues for future clinical investigation. A primary focus should be determining the optimal dosage and treatment duration of ozonated water enemas tailored to various COVID-19 patient profiles. This could involve conducting randomized controlled trials that compare different doses and treatment schedules to ascertain the most efficacious protocol for reestablishing gut equilibrium and reducing the systemic inflammation associated with COVID-19. Additionally, examining the potential synergistic effects of ozonated water enemas in conjunction with other therapeutic approaches, such as probiotics or FMT, may yield superior outcomes in addressing complex gut microbiota imbalances.

## Conclusion

In our study, we endeavoured to emulate the spectrum of systemic pathological responses elicited by SARS-CoV-2 in humans. To this end, we modulated the intestinal flora of mice, thereby compromising their intestinal barrier function. Subsequently, faecal microbiota from critically ill COVID-19 patients was transplanted into these mice, inducing both intestinal and extraintestinal, as well as systemic inflammatory responses. Upon administering ozonated water enemas to the mice, we observed a significant reduction in the expression of caspases and IL-1β. This effect was mediated by activation of the SIRT1-Nrf2/HO-1 pathway, which in turn bolstered the body’s resistance to oxidative stress. The ozonated water enemas exerted a beneficial impact on the intestinal and systemic pathological alterations induced by FMT. These results suggest that ozonated water enemas may serve as a promising clinical therapeutic approach for managing complications and sequelae associated with coronavirus disease.

## Supplementary material

10.1099/jmm.0.002038 Uncited Supplementary Material 1.

## Abbreviations

ACE2: angiotensin-converting enzyme 2
ALT: alanine aminotransferase
ARDS: acute respiratory distress syndrome
AST: aspartate transaminase
ASVs: amplicon sequence variants
COVID-19: coronavirus disease 2019
FMT: faecal microbiota transplantation
HO-1: haem oxygenase-1
IHC: immunohistochemical
IL-1β: interleukin-1β
LEfSe: linear discriminant analysis effect size
Nrf2: nuclear factor erythroid 2-related factor 2
OTUs: Operational Taxonomic Units
PCoA: principal coordinate analysis
ROS: reactive oxygen species
RT-PCR: real-time PCR
SARS-CoV-2: severe acute respiratory syndrome coronavirus 2
SIRT1: silent information regulator of transcription 1
ZO-1: zonula occludens-1

## References

[1] Thangavel H, Dhanyalayam D, Lizardo K, Oswal N, Dolgov E (2023). Susceptibility of fat tissue to SARS-CoV-2 infection in female hACE2 mouse model. IJMS.

[2] Cheng Z, Yang L, Chu H (2022). The gut microbiota: a novel player in autoimmune hepatitis. Front Cell Infect Microbiol.

[3] Dorobisz K, Pazdro-Zastawny K, Misiak P, Kruk-Krzemień A, Zatoński T (2023). Sensorineural hearing loss in patients with long-COVID-19: objective and behavioral audiometric findings. Infect Drug Resist.

[4] Gao Z, Zhang L, Ma J, Sun H, Hu M (2023). Reliability and validity of the Chinese version of the self-directed learning instrument in Chinese nursing students. BMC Nurs.

[5] Akilesh SM, J. R, Palanisamy D, Wadhwani A (2021). Repositioning of drugs to counter COVID-19 pandemic – an insight. CPB.

[6] Wang M, Lin X, Jiao H, Uyanga V, Zhao J (2020). Mild heat stress changes the microbiota diversity in the respiratory tract and the cecum of layer-type pullets. Poult Sci.

[7] Li XM, Lv Q, Chen YJ, Yan LB, Xiong X (2024). Association between childhood obesity and gut microbiota: 16S rRNA gene sequencing-based cohort study. World J Gastroenterol.

[8] Li X, Li C (2018). Analysis of changes in intestinal flora and intravascular inflammation and coronary heart disease in obese patients. Exp Ther Med.

[9] Natarajan A, Zlitni S, Brooks EF, Vance SE, Dahlen A (2022). Gastrointestinal symptoms and fecal shedding of SARS-CoV-2 RNA suggest prolonged gastrointestinal infection. Med.

[10] Vatanen T, Kostic AD, d’Hennezel E, Siljander H, Franzosa EA (2016). Variation in microbiome LPS immunogenicity contributes to autoimmunity in humans. Cell.

[11] Sun Z, Song Z-G, Liu C, Tan S, Lin S (2022). Gut microbiome alterations and gut barrier dysfunction are associated with host immune homeostasis in COVID-19 patients. BMC Med.

[12] Zhou T, Wu J, Zeng Y, Li J, Yan J (2022). SARS‐CoV‐2 triggered oxidative stress and abnormal energy metabolism in gut microbiota. *MedComm*.

[13] Merad M, Martin JC (2020). Pathological inflammation in patients with COVID-19: a key role for monocytes and macrophages. Nat Rev Immunol.

[14] Cheung KS, Hung IFN, Chan PPY, Lung KC, Tso E (2020). Gastrointestinal manifestations of SARS-CoV-2 infection and virus load in fecal samples from a Hong Kong cohort: systematic review and meta-analysis. Gastroenterology.

[15] Agasarov LG, Konchugova TV, Kulchitskaya DB, Davyan OS, Apkhanova TV (2022). Local ozone therapy options for lumbosacral dorsopathy. Eur J Transl Myol.

[16] Clavo B, Santana-Rodríguez N, Llontop P, Gutiérrez D, Suárez G (2018). Ozone therapy as adjuvant for cancer treatment: is further research warranted?. Evid Based Complement Alternat Med.

[17] Galiè M, Covi V, Tabaracci G, Malatesta M (2019). The role of Nrf2 in the antioxidant cellular response to medical ozone exposure. Int J Mol Sci.

[18] Smith NL, Wilson AL, Gandhi J, Vatsia S, Khan SA (2017). Ozone therapy: an overview of pharmacodynamics, current research, and clinical utility. Med Gas Res.

[19] Cenci A, Macchia I, La Sorsa V, Sbarigia C, Di Donna V (2022). Mechanisms of action of ozone therapy in emerging viral diseases: immunomodulatory effects and therapeutic advantages with reference to SARS-CoV-2. Front Microbiol.

[20] Zheng Z, Dong M, Hu K (2020). A preliminary evaluation on the efficacy of ozone therapy in the treatment of COVID-19. J Med Virol.

[21] Rodriguez JAM, Bifano M, Roca Goma E, Plasencia CM, Torralba AO (2021). Effect and tolerability of a nutritional supplement based on a synergistic combination of β-glucans and selenium- and zinc-enriched *Saccharomyces cerevisiae* (ABB C1®) in volunteers receiving the influenza or the COVID-19 vaccine: a randomized, double-blind, placebo-controlled study. Nutrients.

[22] Mozaffari SA, Salehi A, Mousavi E, Zaman BA, Nassaj AE (2022). SARS-CoV-2-associated gut microbiome alteration; a new contributor to colorectal cancer pathogenesis. Pathol Res Pract.

[23] Delgado-Roche L, Riera-Romo M, Mesta F, Hernández-Matos Y, Barrios JM (2017). Medical ozone promotes Nrf2 phosphorylation reducing oxidative stress and pro-inflammatory cytokines in multiple sclerosis patients. Eur J Pharmacol.

[24] Sethi P, Mehan S, Khan Z, Maurya PK, Kumar N (2025). The SIRT-1/Nrf2/HO-1 axis: guardians of neuronal health in neurological disorders. Behav Brain Res.

[25] Liu H, Cheng H, Wang H, Wang Q, Yuan J (2023). Crocin improves the renal autophagy in rat experimental membranous nephropathy via regulating the SIRT1/Nrf2/HO-1 signaling pathway. Ren Fail.

[26] Scuto M, Ontario ML, Salinaro AT, Caligiuri I, Rampulla F (2022). Redox modulation by plant polyphenols targeting vitagenes for chemoprevention and therapy: relevance to novel anti-cancer interventions and mini-brain organoid technology. Free Radical Biology and Medicine.

[27] Kaszubowska L, Kaczor JJ, Karnia MJ, Foerster J, Kmieć Z (2024). Expression of a stress-inducible heme oxygenase-1 in NK cells is maintained in the process of human aging. Front Immunol.

[28] Mosadegh M, Khalkhali A, Erfani Y, Nezamdoost M, Hashemi SH (2024). NBS superfood: a promising adjunctive therapy in critically ill ICU patients with omicron variant of COVID-19. AMB Expr.

[29] Ren K, Yong C, Jin Y, Rong S, Xue K (2025). Unraveling the microbial mysteries: gut microbiota’s role in ulcerative colitis. Front Nutr.

[30] Metcalfe D, Harte AL, Aletrari MO, Al Daghri NM, Al Disi D (2012). Does endotoxaemia contribute to osteoarthritis in obese patients?. *Clinical Science*.

[31] Yu X, Wang Y, Xu Y, Li X, Zhang J (2024). Resveratrol attenuates intestinal epithelial barrier dysfunction via Nrf2/HO-1 pathway in dextran sulfate sodium-induced Caco-2 cells. Immun Inflamm Dis.

[32] Khan S, Wang T, Cobo ER, Liang B, Khan MA (2024). Antioxidative Sirt1 and the Keap1-Nrf2 signaling pathway impair inflammation and positively regulate autophagy in murine mammary epithelial cells or mammary glands infected with *Streptococcus uberis*. Antioxidants.

[33] Aggarwala V, Mogno I, Li Z, Yang C, Britton GJ (2021). Precise quantification of bacterial strains after fecal microbiota transplantation delineates long-term engraftment and explains outcomes. Nat Microbiol.

[34] Staley C, Kaiser T, Beura LK, Hamilton MJ, Weingarden AR (2017). Stable engraftment of human microbiota into mice with a single oral gavage following antibiotic conditioning. *Microbiome*.

[35] Turnbaugh PJ, Ridaura VK, Faith JJ, Rey FE, Knight R (2009). The effect of diet on the human gut microbiome: a metagenomic analysis in humanized gnotobiotic mice. Sci Transl Med.

[36] Eliakim R, Karmeli F, Rachmilewitz D, Cohen P, Zimran A (2001). Ozone enema: a model of microscopic colitis in rats. Dig Dis Sci.

[37] Viebahn-Haensler R, León Fernández OS (2021). Ozone in medicine. The low-dose ozone concept and its basic biochemical mechanisms of action in chronic inflammatory diseases. Int J Mol Sci.

[38] Kuroda K, Azuma K, Mori T, Kawamoto K, Murahata Y (2015). The safety and anti-tumor effects of ozonated water *in vivo*. IJMS.

[39] Yang A, Ding Y, Guo C, Liu C, Xiong Z (2024). PARVB deficiency alleviates cisplatin-induced tubular injury by inhibiting TAK1 signaling. Cell Mol Life Sci.

[40] Torlakov EE (2014). Standardization of negative controls in diagnostic immunohistochemistry: recommendations from the international ad hoc expert panel. Appl Immunohistochem Mol Morphol.

[41] An R, Robbins D, Rey FE, Thibeault SL (2022). Vocal fold mucus layer: comparison of histological protocols for visualization in mice. Laryngoscope Investig Otolaryngol.

[42] Abdul Rashid MR, Syed Mohamad SN, Tajjudin AIA, Roslan N, Jaffar A (2023). COVID-19 pandemic fatigue and its sociodemographic, mental health status, and perceived causes: a cross-sectional study nearing the transition to an endemic phase in Malaysia. IJERPH.

[43] Xanthi Z, Vasiliki P, Stavros A (2022). Apheresis and COVID-19 in intensive care unit (ICU). Transfus Apher Sci.

[44] Cho KH, Kim JE, Bahuguna A, Kang DJ (2023). Ozonated sunflower oil exerted potent anti-inflammatory activities with enhanced wound healing and tissue regeneration abilities against acute toxicity of carboxymethyllysine in zebrafish with improved blood lipid profile. Antioxidants.

[45] Costanzo M, Cisterna B, Vella A, Cestari T, Covi V (2015). Low ozone concentrations stimulate cytoskeletal organization, mitochondrial activity and nuclear transcription. Eur J Histochem.

[46] Fabricius MM, Hitchcock NM, Reuter ZC, Simon ME, Pierce RP (2022). Impact of the COVID-19 pandemic and telehealth implementation in a student-run free clinic. J Community Health.

[47] Haghpanah A, Hosseinpour A, Kallidonis P, Defidio L, Liatsikos E (2023). Are herbal medications, with possible beneficial effects for benign prostatic hyperplasia, better to be continued during the COVID-19 pandemic?. Urologia.

[48] Holmes EA, O’Connor RC, Perry VH, Tracey I, Wessely S (2020). Multidisciplinary research priorities for the COVID-19 pandemic: a call for action for mental health science. Lancet Psychiatry.

[49] Gunnell D, Appleby L, Arensman E, Hawton K, John A (2020). Suicide risk and prevention during the COVID-19 pandemic. Lancet Psychiatry.

[50] Song Y, Liu P, Shi XL, Chu YL, Zhang J (2020). SARS-CoV-2 induced diarrhoea as onset symptom in patient with COVID-19. Gut.

[51] Wang D, Hu B, Hu C, Zhu F, Liu X (2020). Clinical characteristics of 138 hospitalized patients with 2019 novel coronavirus–infected pneumonia in Wuhan, China. JAMA.

[52] Essex M, Millet Pascual-Leone B, Löber U, Kuhring M, Zhang B (2024). Gut microbiota dysbiosis is associated with altered tryptophan metabolism and dysregulated inflammatory response in COVID-19. NPJ Biofilms Microbiomes.

[53] Zhang F, Lau RI, Liu Q, Su Q, Chan FKL (2023). Gut microbiota in COVID-19: key microbial changes, potential mechanisms and clinical applications. Nat Rev Gastroenterol Hepatol.

[54] Xiao F, Tang M, Zheng X, Liu Y, Li X (2020). Evidence for gastrointestinal infection of SARS-CoV-2. Gastroenterology.

[55] Xu Y, Li X, Zhu B, Liang H, Fang C (2020). Characteristics of pediatric SARS-CoV-2 infection and potential evidence for persistent fecal viral shedding. Nat Med.

[56] Lamers MM, Beumer J, van der Vaart J, Knoops K, Puschhof J (2020). SARS-CoV-2 productively infects human gut enterocytes. Science.

[57] Gilbert JA, Blaser MJ, Caporaso JG, Jansson JK, Lynch SV (2018). Current understanding of the human microbiome. Nat Med.

[58] Yeoh YK, Zuo T, Lui GC-Y, Zhang F, Liu Q (2021). Gut microbiota composition reflects disease severity and dysfunctional immune responses in patients with COVID-19. *Gut*.

[59] Reinold J, Farahpour F, Fehring C, Dolff S, Konik M (2021). A pro-inflammatory gut microbiome characterizes SARS-CoV-2 infected patients and a reduction in the connectivity of an anti-inflammatory bacterial network associates with severe COVID-19. Front Cell Infect Microbiol.

[60] Gu S, Chen Y, Wu Z, Chen Y, Gao H (2020). Alterations of the gut microbiota in patients with coronavirus disease 2019 or H1N1 influenza. Clin Infect Dis.

[61] Wang B, Zhang L, Wang Y, Dai T, Qin Z (2022). Alterations in microbiota of patients with COVID-19: potential mechanisms and therapeutic interventions. Sig Transduct Target Ther.

[62] Jeyaraman M, Jeyaraman N, Ramasubramanian S, Balaji S, Nallakumarasamy A (2024). Ozone therapy in musculoskeletal medicine: a comprehensive review. Eur J Med Res.

[63] Hussain S, Sharma DB, Solanki FS, Pathak A, Sharma D (2017). Intraprostatic ozone therapy: a minimally invasive approach in benign prostatic hyperplasia. Urol Ann.

[64] Rapone B, Ferrara E, Qorri E, Inchingolo F, Isola G (2023). Research efficacy of gaseous ozone therapy as an adjuvant to periodontal treatment on oxidative stress mediators in patients with type 2 diabetes: a randomized clinical trial. BMC Oral Health.

[65] Campayo A, Serrano de la Hoz K, García-Martínez MM, Sánchez-Martínez JF, Salinas MR (2019). Spraying ozonated water on Bobal grapevines: effect on grape quality. Food Res Int.

[66] Yu Q, Yang X, Zhang C, Zhang X, Wang C (2020). AMPK activation by ozone therapy inhibits tissue factor‐triggered intestinal ischemia and ameliorates chemotherapeutic enteritis. FASEB j.

[67] Bocci V (2006). Is it true that ozone is always toxic? The end of a dogma. Toxicol Appl Pharmacol.

[68] Li L-J, Yang Y-G, Zhang Z-L, Nie S-F, Li Z (2007). Protective effects of medical ozone combined with traditional Chinese medicine against chemically-induced hepatic injury in dogs. World J Gastroenterol.

[69] Erginel B, Erginel T, Aksoy B, Dokucu Aİ (2014). Effect of ozone therapy (OT) on healing of colonic anastomosis in a rat model of peritonitis. Balkan Med J.

[70] Lacavalla MA, Inguscio CR, Cisterna B, Bernardi P, Costanzo M (2022). Ozone at low concentration modulates microglial activity *in vitro*: a multimodal microscopy and biomolecular study. Microscopy Res & Technique.

[71] Zhang W, Wu M, Chen P, Zhang J, Ma J (2021). Effect of local ozone treatment on rats with anterior rectal resection and the possible mechanisms. Biomed Eng Online.

[72] Chirumbolo S, Franzini M, Tirelli U (2024). Does PI-ME/CFS recall post-COVID (PASC) syndrome?. Virus Res.

[73] Lanza M, Casili G, Torre GLL, Giuffrida D, Rotondo A (2020). Properties of a new food supplement containing *Actinia equina* extract. Antioxidants.

[74] Morishima N, Ogata H, Magae J, Ito Y, Kobayashi J (2023). Analysis method of cellular stress caused by intermediate dose‐rate irradiation using a cell lysate array technique. *Genes to Cells*.

[75] Park J-S, Rustamov N, Roh Y-S (2023). The roles of Nrf2-regulated oxidative stress and mitochondrial quality control in chronic liver diseases. Antioxidants.

[76] Yu X, Wang M, Kong Q (2024). Viral pancreatitis: research advances and mechanisms. Front Microbiol.

[77] Aziz T, Khan AA, Tzora A, Voidarou CC, Skoufos I (2023). Dietary implications of the bidirectional relationship between the gut microflora and inflammatory diseases with special emphasis on irritable bowel disease: current and future perspective. Nutrients.

[78] Jang S-E, Min S-W (2020). Lactobacillus sakei S1 improves colitis induced by 2,4,6-trinitrobenzene sulfonic acid by the inhibition of NF-κB signaling in mice. J Microbiol Biotechnol.

[79] Kaya A, Sonmez M, Kar T, Haholu A, Yildirim Y (2017). Efficiency of ozone therapy in a rat model of experimental uveitis. Ocul Immunol Inflamm.

[80] Liu X-F, Zhou D-D, Xie T, Hao J-L, Malik TH (2018). The Nrf2 signaling in retinal ganglion cells under oxidative stress in ocular neurodegenerative diseases. Int J Biol Sci.

[81] Waugh DT (2019). The contribution of fluoride to the pathogenesis of eye diseases: molecular mechanisms and implications for public health. IJERPH.

[82] Bryan HK, Olayanju A, Goldring CE, Park BK (2013). The Nrf2 cell defence pathway: Keap1-dependent and -independent mechanisms of regulation. Biochem Pharmacol.

[83] Liu X, Peyton K, Ensenat D, Wang H, Hannink M (2007). Nitric oxide stimulates heme oxygenase-1 gene transcription via the Nrf2/ARE complex to promote vascular smooth muscle cell survival. Cardiovasc Res.

[84] Rushworth SA, MacEwan DJ (2011). The role of Nrf2 and cytoprotection in regulating chemotherapy resistance of human leukemia cells. Cancers.

[85] Hariharan N, Maejima Y, Nakae J, Paik J, DePinho RA (2010). Deacetylation of FoxO by Sirt1 plays an essential role in mediating starvation-induced autophagy in cardiac myocytes. Circ Res.

[86] Haigis MC, Sinclair DA (2010). Mammalian sirtuins: biological insights and disease relevance. Annu Rev Pathol.

[87] Qin Z, Song J, Huang J, Jiang S, Zhang G (2023). Mitigation of triptolide-induced testicular Sertoli cell damage by melatonin via regulating the crosstalk between SIRT1 and NRF2. Phytomedicine.

[88] Zhu H, Li X, Qiao M, Sun X, Li G (2023). Resveratrol alleviates inflammation and ER stress through SIRT1/NRF2 to delay ovarian aging in a short-lived fish. J Gerontol A Biol Sci Med Sci.

[89] Li Y, Zhu Y, Hu F, Liu L, Shen G (2023). Procyanidin B2 regulates the sirt1/nrf2 signaling pathway to improve random-pattern skin flap survival. Phytother Res.

[90] Zheng Y, Kou J, Wang P, Ye T, Wang Z (2021). Berberine-induced TFEB deacetylation by SIRT1 promotes autophagy in peritoneal macrophages. *Aging*.

[91] Hong YA, Kim JE, Jo M, Ko GJ (2020). The role of sirtuins in kidney diseases. Int J Mol Sci.

[92] Arioz BI, Tastan B, Tarakcioglu E, Tufekci KU, Olcum M (2019). Melatonin attenuates LPS-induced acute depressive-like behaviors and microglial NLRP3 inflammasome activation through the SIRT1/Nrf2 pathway. Front Immunol.

[93] Park S, Shin J, Bae J, Han D, Park S-R (2020). SIRT1 alleviates LPS-induced IL-1β production by suppressing NLRP3 inflammasome activation and ROS production in trophoblasts. Cells.

[94] Fan J, Zhao Z, Wu H, Fang X, Miao F (2023). Syndecan-3 coregulates milk fat metabolism and inflammatory reactions in bovine mammary epithelial cells through AMPK/SIRT1 signaling pathway. IJMS.

[95] Wang P, Gao C, Guo N, Zhang S-D, Wang W (2018). 2′-O-galloylhyperin isolated from *Pyrola incarnata* Fisch. Attenuates LPS-induced inflammatory response by activation of SIRT1/Nrf2 and inhibition of the NF-κB pathways *in vitro* and *vivo*. Front Pharmacol.

[96] Lee S-I, Min K-S, Bae W-J, Lee Y-M, Lee S-Y (2011). Role of SIRT1 in heat stress- and lipopolysaccharide-induced immune and defense gene expression in human dental pulp cells. J Endod.

[97] Huang W, Shang W, Wang H, Wu W, Hou S (2012). Sirt1 overexpression protects murine osteoblasts against TNF-α-induced injury *in vitro* by suppressing the NF-κb signaling pathway. Acta Pharmacol Sin.

[98] Viebahn-Haensler R, León Fernández OS (2023). Ozone as redox bioregulator in preventive medicine: the molecular and pharmacological basis of the low-dose ozone concept—a review. Int J Mol Sci.

[99] Alimohammadi M, Naderi M (2021). Effectiveness of ozone gas on airborne virus inactivation in enclosed spaces: a review study. Ozone: Sci Eng.

[100] Zheng Y, Li L, Chen B, Fang Y, Lin W (2022). Chlorogenic acid exerts neuroprotective effect against hypoxia-ischemia brain injury in neonatal rats by activating Sirt1 to regulate the Nrf2-NF-κB signaling pathway. Cell Commun Signal.

[101] Abd El-Emam MM, Mostafa M, Farag AA, Youssef HS, El-Demerdash AS (2023). The potential effects of quercetin-loaded nanoliposomes on amoxicillin/clavulanate-induced hepatic damage: targeting the SIRT1/Nrf2/NF-κB signaling pathway and microbiota modulation. Antioxidants.

[102] Sagai M, Bocci V (2011). Mechanisms of action involved in ozone therapy: is healing induced via a mild oxidative stress?. Med Gas Res.

[103] Huang FS, Shah SS, Long S (2023). Principles and Practice of Pediatric Infectious Diseases.

[104] Stojanov M, Baud D, Greub G, Vulliemoz N (2018). Male infertility: the intracellular bacterial hypothesis. New Microbes New Infect.

[105] Zhou H, Huang D, Sun Z, Chen X (2024). Effects of intestinal *Desulfovibrio bacteria* on host health and its potential regulatory strategies: a review. Microbiol Res.

[106] Fraga CG, Croft KD, Kennedy DO, Tomás-Barberán FA (2019). The effects of polyphenols and other bioactives on human health. Food Funct.

[107] Christensen L, Vuholm S, Roager HM, Nielsen DS, Krych L (2019). Prevotella abundance predicts weight loss success in healthy, overweight adults consuming a whole-grain diet ad libitum: a post hoc analysis of a 6-wk randomized controlled trial. J Nutr.

[108] Oñate FP, Chamignon C, Burz SD, Lapaque N, Monnoye M (2023). Adlercreutzia equolifaciens is an anti-inflammatory commensal bacterium with decreased abundance in gut microbiota of patients with metabolic liver disease. IJMS.

[109] Hur KY, Lee MS (2015). Gut microbiota and metabolic disorders. Diabetes Metab J.

[110] Wexler HM (2007). Bacteroides: the good, the bad, and the nitty-gritty. Clin Microbiol Rev.

[111] Russo E (2022). The gut microbiota as a biomarker in epilepsy. Neurobiol Dis.

