# Supplementary material for: Ozone water enema activates SIRT1-Nrf2/HO-1 pathway to ameliorate gut dysbiosis in mice receiving COVID-19 patient-derived faecal microbiota
Source: J Med Microbiol. 2025 Sep 17;74(9):002038. doi: 10.1099/jmm.0.002038 (PMC12444790; doi:10.1099/jmm.0.002038)
Supplement: Uncited Supplementary Material 1. [file jmm-74-02038-s001.pdf]

Supplementary Table 1| Clinical baseline characteristics of all patients selected for fecal transplants to mice in COVID-19 infections.

Characteristic	All Patients case (%)
Total	21
Age	
≤50 years	2（9.52）
51-69 years	5（23.81）
≥70 years	14（66.67）
Sex	
Female	9（42.86）
Male	12（57.14）
Nucleic Acid Test	
Positive	21（100.00）
Negetive	0（0.00）
Symptoms	
Fever	18（85.71）
Continuous Cough	20（95.24）
Shortness of Breath	21（100.00）
Breathing Difficulty	20（95.24）
Respiratory Infection	21（100.00）
Pneumonia	21（100.00）
Blood Gas Analysis	
SaO2 ≤ 90 %	20（95.24）
PaO2 ≤ 60 mmHg	20（95.24）
PaO2/FiO2 ≤ 300mmHg	18（85.71）
Blood Biochemical Indicators	
AST>40 IU/L	21（100.00）
ALT>40 IU/L	21（100.00）
IL-6>7 pg/m l	21（100.00）

*The experimental criteria were as follows: no underlying respiratory disease, COPD; no history of chronic gastroenteritis or gastrointestinal surgery; no mental illness, Parkinson's disease; no diabetes mellitus; and pregnant women were excluded.

Supplementary Table 2 | Metagenome or environmental sample from gut metagenome

Sample	Accession	ID
1	SAMN50547845	50547845
2	SAMN50547846	50547846
3	SAMN50547847	50547847
4	SAMN50547848	50547848
5	SAMN50547849	50547849
6	SAMN50547850	50547850
7	SAMN50547851	50547851
8	SAMN50547852	50547852
9	SAMN50547853	50547853
10	SAMN50547854	50547854
11	SAMN50547855	50547855
12	SAMN50547856	50547856
13	SAMN50547857	50547857
14	SAMN51095753	51095753
15	SAMN51095754	51095754
16	SAMN51095755	51095755
